# Supplementary material for: Genetic Characteristic and RNA-Seq Analysis in Transparent Mutant of Carp–Goldfish Nucleocytoplasmic Hybrid
Source: Genes (Basel). 2019 Sep 12;10(9):704. doi: 10.3390/genes10090704 (PMC6771007; doi:10.3390/genes10090704)
Supplement: Supplementary file 1 [file genes-10-00704-s001.zip › TableS1-S4.pdf]

Table S1. Primer sequences

Gene Bank	Gene Name	Primer (5′→3′)	Product size(bp)
AJ870982	*Gapdh*	AGGGGCTCAGTATGTTGTGG<br>AGGAGGCATTGCTGACAACT	185
M24113.1	*β-actin*	GATGGACTCTGGTGATGGTGTGAC<br>TTTCTCTTTCGGCTGTGGTGGTG	168
XM_019106987.1	*PNP5a*	AGGATTGCCAGGACACAGC<br>TTGGACGGGATAACCCTCATAG	260
XM_019071116.1	*GIMAP8*	ACACGATACTGAGAAGAGAAGC<br>CGCCATTGAGACGCACTT	188
XM_019069292.1	*PNP4a*	GGACTTGCTGGACTGAAC<br>ACGCCTTCTTGGACATAC	155
XM_019088725.1	*EDNRB*	CATCAGTTGGCATCACAGT<br>GAGAGTGTAGAAGATAGCAGTA	338
XM_019098377.1	*URGCP*	AGCGAGGAGCGTTAAGTGT<br>TATCAGTGTTTGGAAGCAGGAG	215
XM_019113351.1	*AKAP12*	ACCTGAAGTAAGTGATGCTGTC<br>ACTAATTGAATGGCTTGCTCTG	201
XM_019070058.1	*GBP1*	GCTTGGCTTCTGCTGACATT<br>ATGCTGCTAAGATGTTGGTGTT	334
XM_019123301.1	*GNA14*	GTTGCTTTAGTTTGTTGCTGGT<br>GTGCCTTGTTGCCTTCCTC	203
XM_019097578.1	*GSLN*	TTTCTTTCTGCTCTTTGCTCTG<br>GGTGTACTCCTCATAACTGTGT	229
XM_019092492.1	*HRG*	AGCACCATCACCACCAGTA<br>GGAGGAAGGACATTGACCAT	184
XM_019116640.1	*APBP2*	TCGGATTCTTCTCGTTCTCATT<br>GCACACAAATACACACCACTC	247

Table S2. Statistic of mRNA data

Sample	Clean Reads Number	Reads Length	Clean Q30 Bases Rate (%)	Mapped reads	Mapping rate (%)
TRCC	63,272,006	145	96.59%	41,800,444	66.06%
RRCC	75,019,864	145	96.71%	50,183,848	66.89%

Table S3. Statistic of miRNA data.

**Sample**	**TRCC**	**RRCC**
Total Raw Reads	10979661	12296112
Total Clean Reads	9876199	11368562
Unique Clean Reads	522171	496650
Total Match Reads	9655021	11263543
Perfect Match Reads	7898353	9385680
Total Clean Reads Mature	6197464	7565200
Unique Clean Reads Mature	2578	2525
Total Clean Reads Novel	552776	725490
Unique Clean Reads Novel	2712	2978

Table S4. DE miRNAs in TM compared to WT

Known miRNA	Fold change	*P*-adjust	Regulation	miRNA	Fold change	*P*-adjust	Regulation
ccr-miR-153b	18.03	1.00E-05	up	ccr-miR-146a	2.01	0.00E+00	up
**Novel miRNA**	**Fold change**	***P*-adjust**	**Regulation**	**miRNA**	**Fold change**	***P*-adjust**	**Regulation**
Novel_410	1383.38	3.27E-94	up	Novel_244	920.59	9.68E-90	down
Novel_435	1241.90	7.97E-87	up	Novel_84	890.06	1.84E-87	down
Novel_87	1029.68	3.26E-75	up	Novel_411	862.58	2.18E-85	down
Novel_341	655.01	7.51E-53	up	Novel_88	851.89	1.40E-84	down
Novel_137	586.89	1.85E-48	up	Novel_104	777.09	1.02E-78	down
Novel_380	356.33	1.63E-32	up	Novel_7	567.93	1.94E-61	down
Novel_392	272.48	3.52E-26	up	Novel_188	389.31	2.39E-45	down
Novel_224	196.50	4.53E-20	up	Novel_293	311.45	8.71E-38	down
Novel_266	170.30	7.55E-18	up	Novel_393	216.79	5.02E-28	down
Novel_155	154.58	1.72E-16	up	Novel_415	174.04	2.15E-23	down
Novel_346	128.38	3.84E-14	up	Novel_62	109.92	6.87E-16	down
Novel_174	120.52	1.95E-13	up	Novel_428	94.65	5.39E-14	down
Novel_427	86.46	3.25E-10	up	Novel_223	87.02	4.88E-13	down
Novel_23	70.74	1.17E-08	up	Novel_395	61.07	1.20E-09	down
Novel_460	62.88	6.90E-08	up	Novel_268	56.49	5.07E-09	down
Novel_459	60.26	1.25E-07	up	Novel_70	54.96	8.15E-09	down
Novel_75	49.78	1.55E-06	up	Novel_288	53.43	1.29E-08	down
Novel_50	47.16	2.91E-06	up	Novel_300	50.38	3.25E-08	down
Novel_157	44.54	5.53E-06	up	Novel_190	48.85	5.27E-08	down
Novel_129	41.92	1.01E-05	up	Novel_391	47.33	8.37E-08	down
Novel_136	41.92	1.01E-05	up	Novel_145	45.80	1.35E-07	down
Novel_273	39.30	1.87E-05	up	Novel_234	41.22	6.03E-07	down
Novel_184	39.30	1.87E-05	up	Novel_315	36.64	2.66E-06	down
Novel_119	34.06	6.62E-05	up	Novel_49	33.59	7.03E-06	down
Novel_160	34.06	6.62E-05	up	Novel_24	33.59	7.03E-06	down
Novel_314	34.06	6.62E-05	up	Novel_74	33.59	7.03E-06	down
Novel_16	31.44	1.22E-04	up	Novel_161	30.53	1.87E-05	down
Novel_202	31.44	1.22E-04	up	Novel_291	29.01	2.94E-05	down
Novel_313	31.44	1.22E-04	up	Novel_290	29.01	2.94E-05	down
Novel_73	31.44	1.22E-04	up	Novel_130	29.01	2.94E-05	down

Table S5. All the DE genes between TM and WT

(Due to the large amount of data, all the differentially regulated genes have been shown in the attached Supplemental Table 5.)
