# Supplementary figures and images for: Genetic Characteristic and RNA-Seq Analysis in Transparent Mutant of Carp–Goldfish Nucleocytoplasmic Hybrid
Source: Genes (Basel). 2019 Sep 12;10(9):704. doi: 10.3390/genes10090704 (PMC6771007; doi:10.3390/genes10090704)

## Supplemental Fig. 1

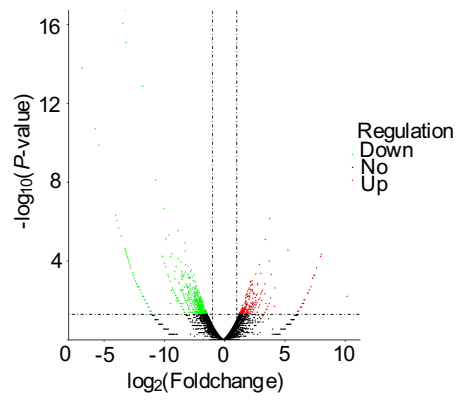

(A)

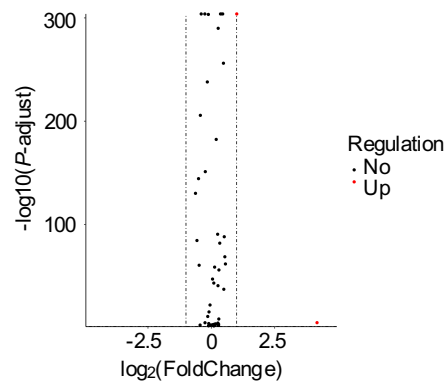

(B)

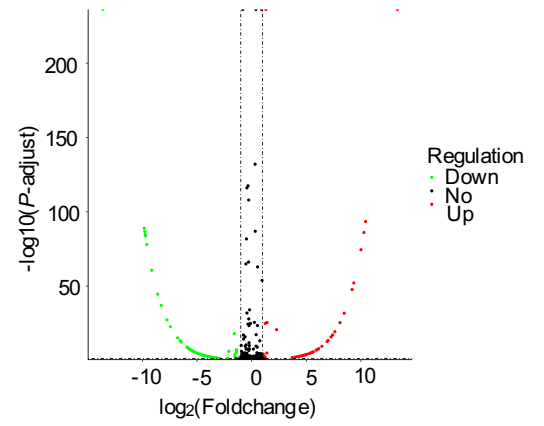

(C)

## Supplemental Fig. 2

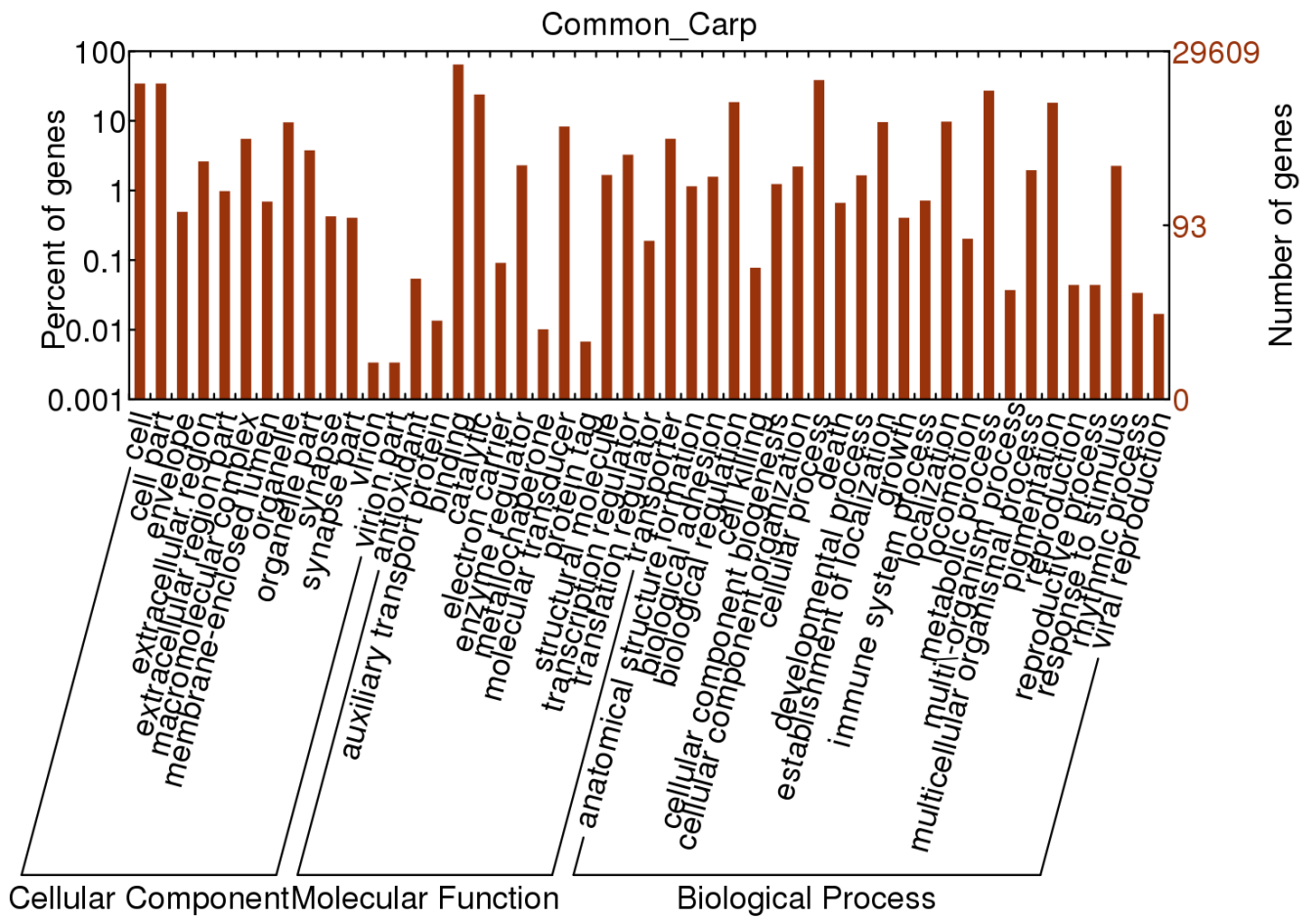

Supplement: Supplementary file 1 [file genes-10-00704-s001.zip › Supplementary Figures.pdf]
